# Supplementary material for: Causal Relationship between Aging and Anorexia Nervosa: A White-Matter-Microstructure-Mediated Mendelian Randomization Analysis
Source: Biomedicines. 2024 Aug 16;12(8):1874. doi: 10.3390/biomedicines12081874 (PMC11351342; doi:10.3390/biomedicines12081874)
Supplement: Supplementary file 1 [file biomedicines-12-01874-s001.zip › Supplementary Figure/Figure S4/Singe SNP plot of MR analysis for TL to ubm-b-1599.pdf]

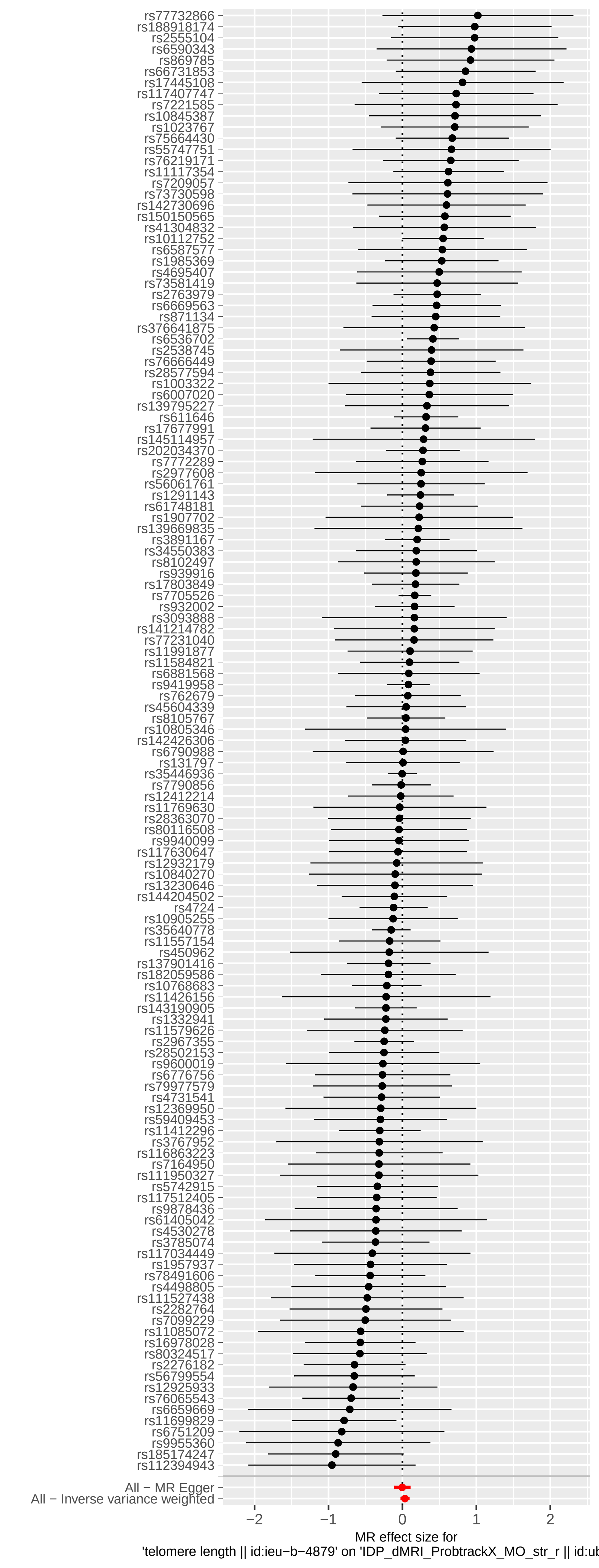
rs77732866
rs188918174
rs2555104
rs6590343
rs869785
rs66731853
rs17445108
rs117407747
rs7221585
rs10845387
rs1023767
rs75664430
rs55747751
rs76219171
rs11117354
rs7209057
rs73730598
rs142730696
rs150150565
rs41304832
rs10112752
rs6587577
rs1985369
rs4695407
rs73581419
rs2763979
rs6669563
rs871134
rs376641875
rs6536702
rs2538745
rs76666449
rs28577594
rs1003322
rs6007020
rs139795227
rs611646
rs17677991
rs145114957
rs202034370
rs7772289
rs2977608
rs56061761
rs1291143
rs61748181
rs1907702
rs139669835
rs3891167
rs34550383
rs8102497
rs939916
rs17803849
rs7705526
rs932002
rs3093888
rs141214782
rs77231040
rs11991877
rs11584821
rs6881568
rs9419958
rs762679
rs45604339
rs8105767
rs10805346
rs142426306
rs6790988
rs131797
rs35446936
rs7790856
rs12412214
rs11769630
rs28363070
rs80116508
rs9940099
rs117630647
rs12932179
rs10840270
rs13230646
rs144204502
rs4724
rs10905255
rs35640778
rs11557154
rs450962
rs137901416
rs182059586
rs10768683
rs11426156
rs143190905
rs1332941
rs11579626
rs2967355
rs28502153
rs9600019
rs6776756
rs79977579
rs4731541
rs12369950
rs59409453
rs11412296
rs3767952
rs116863223
rs7164950
rs111950327
rs5742915
rs117512405
rs9878436
rs61405042
rs4530278
rs3785074
rs117034449
rs1957937
rs78491606
rs4498805
rs111527438
rs2282764
rs7099229
rs11085072
rs16978028
rs80324517
rs2276182
rs56799554
rs12925933
rs76065543
rs6659669
rs11699829
rs6751209
rs9955360
rs185174247
rs112394943
All − MR Egger
All − Inverse variance weighted
−2
−1
0
1
2
MR effect size for
'telomere length || id:ieu−b−4879' on 'IDP_dMRI_ProbtrackX_MO_str_r || id:ub
